# Integrated Phytochemical, Pharmacological, and In Silico Evaluation of the Methanolic Extract of *Cayratia trifolia* Leaves: Antioxidant, Anti‐Inflammatory, and Analgesic Activities

**DOI:** 10.1155/tswj/8390762

**Published:** 2026-06-18

**Authors:** Nasrin Jubayda, Md. Tanveer Ahsan, Md. Kaisar Alam, T. S. Farah Mousumi, Tanbirul Azim Maharaj, Mohammad Arman

**Affiliations:** ^1^ Department of Pharmacy, BGC Trust University Bangladesh, Chittagong, Bangladesh, bgctub-edu.com; ^2^ Department of Pharmacy, University of Chittagong, Chittagong, Bangladesh, cu.ac.bd; ^3^ Department of Pharmacy, International Islamic University Chittagong, Chittagong, Bangladesh, iiuc.ac.bd; ^4^ Department of Pharmacy, Jahangirnagar University, Dhaka, Bangladesh, juniv.edu

**Keywords:** ADME/T, analgesic, anti-inflammatory, antioxidant, *Cayratia trifolia*, in vitro, in vivo, molecular docking, phytochemicals

## Abstract

*Cayratia trifolia* is traditionally used for treating pain, inflammation, and oxidative disorders, yet comprehensive experimental and computational validation of its pharmacological potential remains limited. The aim of this study is to investigate the phytochemical profile and evaluate antioxidant, anti‐inflammatory, and analgesic activities of methanolic and n‐hexane fractions of *C. trifolia* leaves using integrated in vitro, in vivo, and in silico approaches. Leaves were extracted with methanol and fractionated via a modified Kupchan method. Phytochemical screening, DPPH radical scavenging, HRBC membrane stabilization, carrageenan‐induced paw edema, acetic acid‐induced writhing, and tail immersion assays were performed. In silico analysis included PASS prediction, ADME/T profiling, and molecular docking of 40 previously documented GC–MS compounds against COX‐2 (PDB: 6COX, 1CX2, 5IKR, and 1CQE) and CYP2C9 (PDB: 1OG5 and 4L7B) protein targets. Methanolic extract demonstrated potent antioxidant activity (IC_50_ = 2.69 * μ*g/mL) and 87.46% HRBC membrane stabilization. Significant anti‐inflammatory effects were observed in vivo through reduced paw edema. The n‐hexane fraction produced notable analgesic responses, inhibiting writhing by 21.84% and increasing reaction time in the tail immersion test by 59.44% ( ^∗∗∗∗^ *p* < 0.0001). In silico analyses identified multiple bioactive compounds—especially 2‐cyclohexylethyl isobutyl ester and lup‐20(29)‐en‐3‐yl acetate—with binding affinities surpassing diclofenac, aspirin, and ascorbic acid, along with favourable ADME/T profiles. The combined experimental and computational findings indicate that *C. trifolia* contains phytoconstituents with strong therapeutic potential. Its methanolic fraction, supported by in silico validation, represents a promising source for developing natural antioxidant, anti‐inflammatory, and analgesic lead molecules.

## 1. Introduction

Medicinal plants have been integral to global healthcare since ancient civilizations, and they continue to play a prominent role in contemporary medicine. Approximately 85% of the world′s population still relies on herbal remedies for primary healthcare needs, highlighting their long‐standing therapeutic value [1]. For millennia, humans have depended on plants for healing purposes, recognizing their ability to alleviate symptoms and restore health [2]. A medicinal plant is defined by its capacity to elicit therapeutic or pharmacological effects in the human body, largely due to its diverse secondary metabolites. These bioactive constituents are known to reduce pain, facilitate wound healing, and exert a wide array of beneficial physiological actions. Consequently, medicinal plants serve as rich reservoirs for the discovery and development of novel pharmacological agents.

*Cayratia trifolia* Domin Syn. Linn—commonly known as *Vitis trifolia* (family: Vitaceae) or fox grape—is one such plant with extensive ethnomedicinal relevance. It is referred to as Amlabel and Ramchana in Hindi and Amlavetash in Sanskrit. The species is native to Australia and widely distributed across Asia and India. It grows as a perennial climbing herb with distinctive trifoliate leaves. In the Philippines, where it thrives at low elevations, it is locally termed “kalit‐kalit.” Its distribution ranges across southern China, India, the Moluccas, the Caroline Islands, and throughout the Malayan region [3, 4]. In India, it grows profusely in West Bengal, Assam, Tripura, Rajasthan, and Jammu [5]. Beyond South Asia, the plant extends across Bangladesh, Burma, Ceylon, Cambodia, Indonesia, Laos, Malaysia, Malacca, Pakistan, Thailand, and Vietnam [6, 7].

The entire plant is valued in traditional medicine for its diuretic properties and is used to manage tumors, neuralgia, splenopathy, leucorrhea, and conditions requiring an astringent effect [8]. Leaves, roots, and seeds are often prepared as poultices to treat ulcers and boils. The sap of the stems and the juice of the leaves are used as aphrodisiacs. Root extracts are traditionally employed to treat anemia, gastrointestinal ailments, and snake bites, while root paste is applied to caruncles and inflammatory skin lesions [9]. Roots mixed with black pepper are used as compresses for boils, and root paste combined with coconut oil is administered as a decoction for 3 consecutive days. Leaf paste is additionally used in managing eczema [10]. These practices indicate the plant′s potential anti‐inflammatory, antimicrobial, and wound‐healing effects.

Oxidative stress and inflammation, both central to many chronic diseases, may underpin several of the traditional uses of *C. trifolia*. Free radicals contribute to cellular damage and are known triggers of inflammatory processes. Although oxidation is a natural biochemical phenomenon essential to metabolism, uncontrolled oxidative activity leads to oxidative stress, which plays a pivotal role in aging and the onset of chronic illnesses. Therefore, numerous studies have sought natural antioxidant compounds to mitigate oxidative damage and prevent associated diseases [11]. Inflammation itself is the body′s defensive response to harmful stimuli such as pathogens, toxins, or tissue injury. While essential for healing, persistent or uncontrolled inflammation contributes to tissue destruction and the progression of disorders such as arthritis and autoimmune diseases.

Nonsteroidal anti‐inflammatory drugs (NSAIDs), including indomethacin and ibuprofen, are commonly used to manage both acute and chronic inflammation [12]. Similarly, nonopioid analgesics like aspirin are widely used for pain relief. These medications are cornerstones in treating conditions such as rheumatoid arthritis. However, long‐term use is associated with adverse effects such as gastrointestinal irritation, renal damage, respiratory depression, and the potential for dependence. These concerns highlight the need for safer, plant‐derived alternatives capable of modulating inflammatory and pain pathways with fewer side effects.

Modern pharmaceutical development is a complex and resource‐intensive process, but recent advances in computational technologies have significantly accelerated drug discovery. Computer‐aided drug design (CADD) has become an indispensable tool in identifying promising therapeutic candidates more efficiently and cost‐effectively [13]. By simulating molecular interactions and predicting biological activity, CADD reduces reliance on animal testing, enhances safety profiling, and aids in discovering new applications for existing drugs. It plays a crucial role in supporting medicinal chemists and pharmacologists throughout the drug development pipeline [14].

Although earlier findings report antimicrobial, antifungal, antiprotozoal, hypoglycemic, anticancer, and diuretic activities of *C. trifolia*, comprehensive research that brings together laboratory, animal, and computational evaluations is still insufficient. Its possible antioxidant, anti‐inflammatory, and analgesic properties, which align with its long history of traditional use, have not yet been confirmed through an integrated experimental and digital analysis. For this reason, well‐structured scientific studies are necessary to validate its ethnomedicinal applications and to determine its value as a promising source of new therapeutic compounds.

## 2. Materials and Methods

### 2.1. Plant Collection and Extraction

Fresh leaves of *C. trifolia* were collected from the Hazarikhil Wildlife Sanctuary, Chittagong, Bangladesh, and taxonomically confirmed by a specialist from the University of Chittagong. The leaves were washed, shade‐dried for 14 days, and powdered. A total of 450 g of leaf powder was macerated in 2.5 L of methanol for 7 days with occasional shaking. The filtrate was evaporated at 50°C using a rotary evaporator to obtain a crude methanolic extract of *C. trifolia* (MECT). Subsequent fractionation following a modified Kupchan protocol yielded the n‐hexane fraction (n‐hexane extract of *C. trifolia* [NHCT]). Further fractionation of the methanolic extract was performed to separate constituents by polarity and to evaluate whether pharmacological activities were enriched in the less polar n‐hexane fraction.

### 2.2. Phytochemical Screening

Qualitative phytochemical testing was conducted using various methods, including the ferric chloride and lead acetate test for phenols, the Wagner and Mayer tests for alkaloids, the Salkowski test for triterpenes, the Liebermann–Burchard and Salkowski reaction tests for steroids, the acetone–water test for resins, the sodium hydroxide reagent test for glycosides, the Keller–Killiani test for glycosides, the shake or foam test for saponins, the general test for resins, Fehling′s test for reducing sugars, the Molisch′s test for carbohydrates, the ferric chloride test and lead subacetate test for tannins, the Biuret test and the xanthoproteic test for proteins, and the general test and spot test for fats and fixed oils [15].

### 2.3. In Vitro Antioxidant Activity

Using the procedure described in Rohmah et al. [16], the antioxidant potential of MECT was evaluated by examining the free radical quenching effect of DPPH. A freshly prepared 0.004% (*w*/*v*) DPPH solution was made by dissolving DPPH in analytical‐grade methanol. The solution was protected from light and was used immediately to prevent degradation. The MECT was dissolved in methanol to obtain a stock solution. From this stock, serial dilutions were prepared to yield final concentrations of 500, 250, 125, 62.5, 31.25, and 15.625 *μ*g/mL. For each concentration, 2 mL of the extract solution was mixed with 3 mL of the freshly prepared DPPH solution in clean test tubes and incubated at room temperature for 30 min. A control solution was prepared by mixing 2 mL of methanol with 3 mL of the DPPH solution, without the extract. Methanol was used as the blank. After incubation, the decrease in absorbance of each reaction mixture was measured at 517 nm using a UV–visible spectrophotometer. This expression was employed to determine the percent inhibition of the DPPH:

%Scavenging activity=Ac−AsAc×100

where *A*_*c*_ is the absorbance of the control and *A*_*s*_ is the absorbance of the sample/standard.

### 2.4. In Vitro Anti‐Inflammatory Activity

The hypotonicity‐induced hemolysis method to assess the extract′s anti‐inflammatory qualities [17]. Fresh human blood was collected from a healthy volunteer who had not consumed any anti‐inflammatory or analgesic drugs for at least 2 weeks prior to blood collection. The collected blood was immediately mixed with an equal volume of Alsever′s solution to prevent coagulation. The packed red blood cells were washed three times with isotonic saline solution (0.9% NaCl) until a clear supernatant was obtained. After washing, a 10% (*v*/*v*) human HRBC (HRBC) suspension was prepared by resuspending the cells in isotonic saline, and the leaf extract was dissolved in an appropriate solvent to prepare a stock solution. From this stock, working solutions of different concentrations (2000, 1000, 500, 250, and 125 *μ*g/mL) were prepared. Diclofenac sodium was used as the standard anti‐inflammatory drug and was prepared at the same concentrations.

The control sample was prepared using distilled water instead of the extract or standard drug while maintaining all other components at the same volumes.

The supernatant obtained after centrifugation was carefully transferred into cuvettes, and the absorbance was measured at 560 nm using a UV–visible spectrophotometer.

Each test tube was then filled by adding 0.5 mL suspended HRBC, 1 mL phosphate buffer solution, and 2 mL hypotonic saline. After that, the test tubes underwent incubation for 30 min at 37°C. Then, the tubes underwent cooling and centrifugation for 20 min at 4000 rpm. Absorbance of the supernatant solution was then assessed with a wavelength set to 560 nm using a UV spectrophotometer.

Percent inhibition of protein denaturation has been measured by this expression:

Ac−AsAc×100

where *A*_*c*_ is the absorbance of the control and *A*_*s*_ is the absorbance of the sample/standard.

### 2.5. Experimental Animal

For this study, *Swiss albino* mice of both genders with a body weight ranging from 25 to 35 g and between 2 and 2.5 months old were employed. All the animals were obtained from Rajshahi. They were housed in the Animal House at the University of Chittagong′s Pharmacy Department in an experimental setup maintained with a 12 h light and dark regimen, 25°C (±2°C) with 45% (±5%) relative humidity. The animals have been allowed continuous access to water and a typical laboratory diet. Twelve hours prior to and throughout the trial, food was removed. These animals are housed in the test environment for at least 3–4 days before the test since they are extremely sensitive to changes in their surroundings. The study was reviewed and was authorized by the Pharmacy Department′s P&D Committee (Pharm‐P&D‐61/08‐16–122) at the International Islamic University Chittagong, Bangladesh.

### 2.6. In Vivo Anti‐Inflammatory Activity Using Carrageenan‐Induced Paw Edema Test

Carrageenan has been employed in this experiment for the induction of paw edema in the right posterior limb of mice [18]. Four mouse groups, each consisting of five mice, received 400 mg/kg of methanolic and n‐hexane extracts orally, 10 mL/kg of a 1% Tween 80 solution, and 10 mg/kg of diclofenac sodium intraperitoneally. The right hind paw received 100 *μ*L of 1% weight by volume carrageenan prepared in 0.9% saline solution via subcutaneous injection 1 h after extracts were administered and half an hour after the positive control. A digital vernier caliper was employed in measuring the circumference of the paws prior to the carrageenan injection and at 1, 2, 3, and 4 h later. The data were compared to preinjection values and represented as the variation in paw thickness (millimeters).

The inhibition rate of edema expressed as a percentage has been measured by this expression:

Inhibition %=Ct−C0 control−Ct−C0 treatedCt−C0 control×100

Here, *C*_*t*_ is the mean paw circumference for each group at different time intervals and *C*_0_ is the mean paw circumference for each group before carrageenan injection.

### 2.7. In Vivo Analgesic Activity

#### 2.7.1. Acetic Acid–Induced Writhing Test

While monitoring the writhing response, characterized by abdominal muscle contractions and hind limb extension after intraperitoneal acetic acid injection, the peripheral analgesic effect was investigated [19]. A total of 20 experimental mice were selected at random and categorized into four groups. Forty minutes before a dose of 0.7% acetic acid (0.1 mL for every 10 g of body weight) delivered intraperitoneally, animals were given the negative control, which consisted of normal saline with 1% Tween 80 (0.1 mL/10 g of body weight), whereas diclofenac sodium was utilized for the positive control group at 100 mg/kg, and MECT and NHCT were administered at 400 mg/kg. The 40‐min time has been chosen to guarantee that the given samples will be properly absorbed. After administering acetic acid for 5 min, each mouse′s writhing was then recorded for 15 min. As a measure of analgesia, the inhibition of writhing, expressed as a percentage relative to the negative control, was computed with the help of the expression in the following:

%of inhibition=Nc−NtNc×100

Here, *N*_*c*_ is the number of writhes in the control group and *N*_*t*_ is the number of writhes in the test group.

#### 2.7.2. Tail Immersion Test

The central mechanism of pain relief was assessed through the tail immersion method. In this test, pain responses in animals were induced using a thermal stimulus by placing the tail′s tip in heated water [20]. Twenty male mice have been evenly distributed among four experimental groups consisting of five mice each. Five centimeters of the tail portion was submerged in a water bath held at 55°C ± 0.5°C, and the duration until the tail was quickly pulled back by the mouse, defined as the reaction time, was measured, with a maximum cut‐off of 20 min. Thirty minutes before treatment, the baseline reaction time was determined. After that, all groups of mice were given methanolic extract and n‐hexane extract at 400 mg/kg concentrations, diclofenac sodium at 2 mg/kg body weight, and a negative control. Response times were then measured at 30, 60, 90, and 120 min after treatment. The percentage increase in the length of time the tail was immersed was determined in comparison to the control at the same time interval. Higher elongation percentages reflect enhanced central analgesic activity. Test samples were assessed for central analgesic activity relative to the standard drug.

Elongation %=Latency of test−Latency of controlLatency of test×100

### 2.8. Statistical Analysis

Outcomes of statistical analysis are shown in the form of mean ± SEM (standard error of the mean) for five animals. Version 8.4.2 of GraphPad Prism (GraphPad Software Inc., San Diego, CA) was employed for data analysis. Dunnett′s *t*‐test has been performed to conduct the one‐way analysis of variance (ANOVA) to estimate the degree of statistical significance. Statistical significance is attributed to *p* values less than 0.05, 0.01, 0.001, and 0.0001. An unpaired *t*‐test was used to assess differences between the test groups and the control group in order to identify statistically significant differences in this study.

### 2.9. In Silico Study

#### 2.9.1. Software and Tools

PubChem, MGL Tools, Swiss‐pdbViewer (v4.1), PyRx, AutoDock Vina, Discovery Studio Visualiser 2025 (BIOVIA), and the Protein Data Bank (PDB) were utilized for the analysis.

#### 2.9.2. Selection of Ligands

The ligands selected for this study were derived from previously reported gas chromatography–mass spectrometry (GC–MS) analyses of *C. trifolia* leaf extracts, as documented in the literature [21]. All identified phytoconstituents were retrieved from the PubChem database in SDF format to enable standardized preparation for computational evaluation. Selection of the compounds was guided by their relative abundance in the GC–MS profile as well as their documented biological relevance, particularly in relation to antioxidant, anti‐inflammatory, and analgesic activities. These ligands were then subjected to molecular docking analyses to investigate their potential interactions with the chosen protein targets associated with the therapeutic pathways under investigation.

#### 2.9.3. Prediction of Activity Spectra for Substances (PASS)

The potential biological impact of 40 sample compounds from *C. trifolia* leaf extract was analyzed using the PASS online tools. The Pa and Pi values span from 0.000 to 1.000. The substance is anticipated to exhibit biological activity when its Pa value crosses its Pi value. A Pa value of 0.7 signifies robust biological activity, values ranging from 0.5 to 0.7 imply modest therapeutic potential, and values below 0.5 denote little or negligible pharmacological action [22].

#### 2.9.4. Absorption, Distribution, Metabolism, Excretion, and Toxicity (ADME/T) and Drug‐Likeness Profiling

To assess the pharmacokinetic suitability and safety of the selected phytoconstituents, ADME/T analyses were conducted using the pKCSM, SwissADME, and ADMETsar 2.0 platforms. These computational tools provide predictive models for oral absorption, blood–brain barrier (BBB) permeability, metabolic stability, clearance, and toxicity. Drug‐likeness was evaluated according to Lipinski′s rule of five and other physicochemical filters relevant to oral bioavailability. Compounds demonstrating acceptable absorption characteristics, favorable distribution patterns, low predicted toxicity, and compliance with drug‐likeness criteria were prioritized for docking studies. This screening ensured the selection of ligand candidates with both therapeutic potential and appropriate pharmacokinetic behavior [23].

#### 2.9.5. Validation of Ligands

Prior to docking, the physicochemical and pharmacokinetic profiles of the shortlisted phytochemicals were further validated using the pKCSM online tool. Parameters such as aqueous solubility, intestinal absorption, metabolic liability, and predicted toxicity were examined to ensure suitability for interaction with therapeutic targets. Only compounds demonstrating favorable ADME/T characteristics and acceptable drug‐likeness scores were retained for subsequent molecular docking. This validation step was essential to ensure that the ligands possessed plausible biological and pharmacokinetic attributes for therapeutic development [24].

#### 2.9.6. Protein Preparation

Proteins associated with antioxidant, anti‐inflammatory, and analgesic pathways were obtained from the RCSB Protein Data Bank. Human cyclooxygenase‐2 (COX‐2; PDB: 5F19, resolution 2.4 Å) was chosen for analgesic action, and celecoxib was used as a cocrystallized ligand to help find the active site. For anti‐inflammatory action, human COX‐2 (PDB: 5IKR, resolution 2.4 Å) was employed. Two different proteins were used to test for antioxidant activity: human cytochrome P450 2C9 (CYP2C9; PDB: 1OG5) and Kelch‐like ECH‐associated protein 1 (Keap1; PDB: 4L7B). Keap1 is a negative regulator of the transcription factor Nrf2. When Nrf2 is in its normal state, Keap1 helps it break down in the proteasome. Inhibition of Keap1 disrupts the Keap1–Nrf2 protein–protein interaction, facilitating Nrf2′s translocation to the nucleus and the activation of antioxidant response element (ARE)–driven genes, such as heme oxygenase‐1 (HO‐1) and NAD(P)H quinone oxidoreductase 1 (NQO1). This elucidates the mechanistic basis for its application as a computational antioxidant target. Based on previous research and structural annotations [25, 26]. Under the GROMOS96 force field, subsequent energy minimization of protein structures was carried out in Swiss‐PdbViewer using conjugate gradient and steepest‐descent techniques. Before docking, ligand structures were individually energy‐minimized in the PyRx environment using the MMFF94 force field, which is suitable for small organic compounds [27, 28].

#### 2.9.7. Molecular Docking

Molecular docking was performed using AutoDock Vina implemented within the PyRx 0.3 interface. The docking procedure explored ligand–protein interactions within the predefined active sites of each target protein. The prepared ligands were energy‐minimized and converted to appropriate file formats, and molecular docking was conducted via AutoDock Vina integrated into the PyRx 0.3 interface. The synthesized ligands were placed under energy minimization, transformed into PDBQT format, and docked in the designated active site of each target protein utilizing specified grid box parameters. The grid box for human COX‐2 (PDB: 5F19, analgesic activity) was centered at coordinates *X* = 28.2492, *Y* = 31.0890, *Z* = 65.2578 Å, with uniform dimensions of 25.0 × 25.0 × 25.0 Å. The grid box for human COX‐2 (PDB: 5IKR, anti‐inflammatory activity) was centered at coordinates *X* = 38.0529, *Y* = 2.4453, *Z* = 61.1920 Å, with uniform dimensions of 25.0 × 25.0 × 25.0 Å. The grid box for CYP2C9 (PDB: 1OG5, antioxidant activity) was centered at coordinates *X* = 20.0226, *Y* = 88.5511, *Z* = 39.0260 Å, with uniform dimensions of 25.0 × 25.0 × 25.0 Å. The grid box for Keap1 (PDB: 4L7B, antioxidant activity) was centered at *X* = 3.3410, *Y* = 2.6644, *Z* = 28.5826 Å, with uniform dimensions of 25.0 × 25.0 × 25.0 Å. An exhaustiveness value of 8 was utilized for all docking runs. Binding affinities, measured in kilocalories per mole, and interaction characteristics were recorded for each ligand. The binding conformations and chemical interactions, encompassing hydrogen bonding, hydrophobic interactions, pi–pi stacking, and pi–sigma effects, were visualized using BIOVIA Discovery Studio Visualizer 2025. Following PASS screening of 40 compounds, the 20 candidates exhibiting the highest Pa values were selected for docking studies. Binding affinities (kilocalories per mole) and interaction profiles were recorded for each ligand. Binding poses and molecular interactions—such as hydrogen bonding, hydrophobic contacts, *π*–*π* stacking, and cation–*π* effects—were visualized using PyMOL and BIOVIA Discovery Studio Visualizer 2025 [29]. A total of 40 compounds underwent PASS screening, from which the Top 20 candidates were initially selected based on Pa values; however, following ADME/T screening, compounds flagged for AMES mutagenicity, hepatotoxicity, or failure to meet drug‐likeness criteria were excluded, resulting in 18 compounds for analgesic and 17 compounds each for anti‐inflammatory and antioxidant docking analyses.

## 3. Results

### 3.1. Phytochemical Screening

Primary phytochemical evaluation of MECT leaves confirmed alkaloids, tannins, triterpenes, phytosterols, flavonoids, saponins, carbohydrates, proteins, fats, and fixed oils, which give the plant its medicinal potency against certain diseases, can be seen in Table 1.

**Table 1:** Phytochemical screening of the methanolic extract of *C. trifolia*.

Sl. no.	Phytochemicals	Test	Result
1	Alkaloids	Mayer′s test	+
		Wagner′s test	+

2	Steroids	Salkowski′s reaction test	−
		Liberman–Burchard test	−

3	Tannins	Ferric chloride test	+
4	Triterpenes	Salkowski′s reaction test	+
5	Phytosterols	Salkowski′s reaction test	+

6	Flavonoids	Zn–HCl reduction test	+
		Lead acetate test	+

7	Saponins	Shake or foam test	+
		Foam test	+
8	Resins	Turbidity test	−
9	Glycosides	NaOH reagent test	−
10	Cardiac glycosides	Keller–Killiani test	−
11	Anthraquinone glycoside	Hydroxy anthraquinone test	−

12	Phenols	Ferric chloride test	−
		Lead acetate test	−

13	Reducing sugars	Fehling′s test	−
		Benedict′s test	−
14	Carbohydrates	Molisch′s test	+

15	Proteins	Biuret test	+
		Xanthoproteic test	+
16	Fats and fixed oils	Spot test	+

*Note:* + indicates presence and − indicates absence.

### 3.2. In Vitro Antioxidant Activity

**Figure 1:**
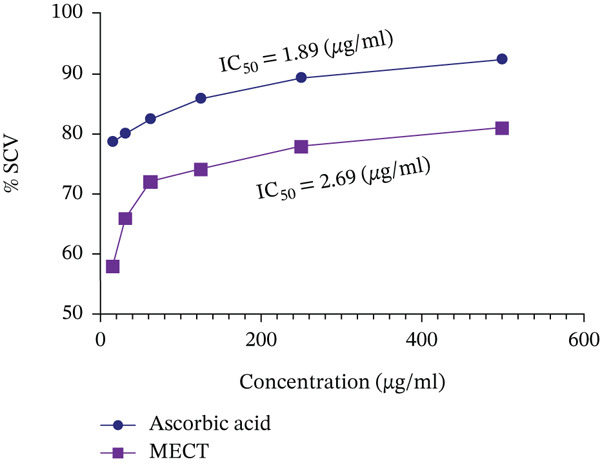
Calibration curve of ascorbic acid and MECT for the DPPH free radical scavenging. MECT, methanolic extract of *Cayratia trifolia.*

According to Figure 1, at a 500 *μ*g/mL solution concentration, MECT and AA showed radical quenching effects of 81.099% and 92.44%, accordingly. The IC_50_ data for both the extract and AA has been recorded as 2.69 and 1.89 *μ*g/mL, as stated.

### 3.3. In Vitro Anti‐Inflammatory Activity

**Figure 2:**
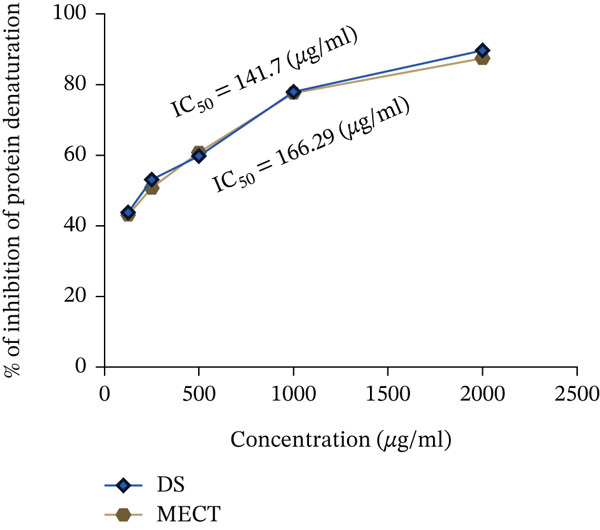
Calibration curve of diclofenac sodium and MECT for the HRBC membrane stabilization method. DS, diclofenac sodium; MECT, methanolic extract of *Cayratia trifolia.*

Figure 2 presents that the extract shows maximum inhibition (87.46%) regarding the HRBC membrane at a concentration of 2000 *μ*g/mL solution. MECT exhibits strong anti‐inflammatory potency, characterized by an IC_50_ of 166.29 *μ*g/mL in comparison to diclofenac sodium, which shows 89.69% inhibition and an IC_50_ value of 141.7 *μ*g/mL.

### 3.4. In Vivo Anti‐Inflammatory Activity Using Carrageenan‐Induced Paw Edema Test

**Figure 3:**
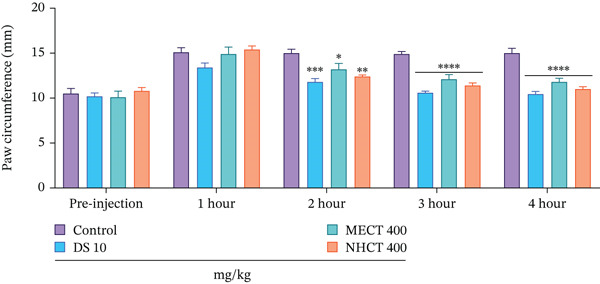
Screening of anti‐inflammatory activity of crude methanolic extract and its soluble fraction of *C. trifolia* leaves by calculating the mean paw circumference using the carrageenan‐induced paw edema test. Values were represented as mean ± SEM (*n* = 5);  ^∗∗∗∗^ *p* < 0.0001,  ^∗∗∗^ *p* < 0.001,  ^∗∗^ *p* < 0.01, and  ^∗^ *p* < 0.05 are statistically significant compared to control, followed by Dunnett′s test (GraphPad Prism 8.4). DS, diclofenac sodium; MECT, methanolic extract of *Cayratia trifolia*; NHCT, n‐hexane extract of *Cayratia trifolia.*

In this investigation, no reduction of paw edema caused by carrageenan was observed for the control, while diclofenac sodium, which served as the reference standard, significantly reduced paw edema starting from the second hour. Edema was decreased by MECT and NHCT in comparison with the control group. The n‐hexane extract administered at 400 mg/kg of body weight showed the greatest effect following 2 and 3 h (Figure 3).

### 3.5. In Vivo Analgesic Activity

#### 3.5.1. Acetic Acid–Induced Writhing Test

During this investigation, the control group exhibited the highest number of writhes, whereas the reference standard showed the lowest number and produced a significant 60.19% inhibition of writhing. In contrast, MECT 200 and MECT 400 produced 36.89% and 21.84% inhibition, respectively, as shown in Table 2.

**Table 2:** Analgesic activity of MECT and NHCT (acetic acid–induced writhing test).

Treatment group (mg/kg)	Number of writhing (*m* *e* *a* *n* ± *S* *E* *M*)	% inhibition of writhing
1% Tween 80	41.2 ± 1.24	—
DS 10	16.4 ± 1.03^∗∗∗∗^	60.19
MECT 400	26 ± 0.83^∗∗∗∗^	36.89
NHCT 400	32.2 ± 0.97^∗∗∗∗^	21.84

*Note:* Values were represented as mean ± SEM. Values were represented as mean ± SEM (*n* = 5).

Abbreviations: DS, diclofenac sodium; MECT, methanolic extract of *Cayratia trifolia*; NHCT, n‐hexane extract of *Cayratia trifolia*.

^∗∗∗∗^ *p* < 0.0001 is statistically significant compared to control, followed by Dunnett′s test (GraphPad Prism 8.4).

#### 3.5.2. Tail Immersion Test

NHCT produced 64.07%, 62.53%, 63.98%, and 59.44% elongation of reaction time at 30, 60, 90, and 120 min, respectively, whereas MECT produced 56.29%, 57.85%, 53.22%, and 49.12% elongation at the corresponding time points, as shown in Table 3. Although MECT demonstrated a significant effect, its elongation of reaction time was lower than that of NHCT. Overall, NHCT exhibited the strongest central analgesic response among the extracts.

**Table 3:** Analgesic activity of MECT and NHCT (tail immersion test).

Treatment group (mg/kg)	(% of elongation)				
	Pretreatment	30 min	60 min	90 min	120 min
1% Tween 80	3.71 ± 0.16	3.72 ± 0.15	3.61 ± 0.13	3.40 ± 0.24	3.41 ± 0.25
DS 10	3.45 ± 0.18	11.81 ± 0.34^∗∗∗∗^ (68.43%)	10.11 ± 0.69^∗∗∗∗^ (64.34%)	10.08 ± 0.10^∗∗∗∗^ (66.24%)	9.24 ± 0.21^∗∗∗∗^ (62.91%)
MECT 400	3.79 ± 0.18	8.53 ± 0.23^∗∗∗∗^ (56.29%)	8.56 ± 0.19^∗∗∗∗^ (57.85%)	7.27 ± 0.12^∗∗∗∗^ (53.22%)	6.73 ± 0.28^∗∗∗∗^ (49.12%)
NHCT 400	3.86 ± 0.55	10.37 ± 0.41^∗∗∗∗^ (64.07%)	9.62 ± 0.15^∗∗∗∗^ (62.53%)	9.45 ± 0.57^∗∗∗∗^ (63.98%)	8.45 ± 0.25^∗∗∗∗^ (59.44%)

*Note:* The data were represented as mean ± SEM. Values were represented as mean ± SEM (*n* = 5).

Abbreviations: DS, diclofenac sodium; MECT, methanolic extract of *Cayratia trifolia*; NHCT, n‐hexane extract of *Cayratia trifolia.*

^∗∗∗∗^ *p* < 0.0001 is statistically significant compared to control methanolic extract of *Cayratia trifolia* and n‐hexane extract of *Cayratia trifolia*, followed by Dunnett′s test (GraphPad Prism 8.4).

### 3.6. PASS Prediction Analysis

The in silico study for the 40 compounds from *C. trifolia* leaf extract revealed promising pharmacological potential in Table 4. Based on their Pa (probability of being active) and Pi (probability of being inactive) values, the PASS prediction analysis revealed that the compounds have significant antioxidant, anti‐inflammatory, and analgesic properties. The minimum limit for a chemical to be considered physiologically active was set at Pa > 0.3. According to this requirement, 40 compounds showed anticipated analgesic efficacy, 38 compounds showed predicted anti‐inflammatory activity, and 39 compounds showed predicted antioxidant activity. The most robust predicted pharmacological potential was shown by compounds with Pa > 0.7, such as lup‐20(29)‐en‐3‐yl acetate (anti‐inflammatory Pa = 0.737; analgesic Pa = 0.679) and 11,14,17‐eicosatrienoic acid methyl ester (antioxidant Pa = 0.887; anti‐inflammatory Pa = 0.804).

**Table 4:** PASS prediction value of compounds from *Cayratia trifolia* leaf extract for antioxidant, anti‐inflammatory, and analgesic activities.

S. no.	Compound name	Antioxidant		Anti‐inflammatory		Analgesic	
		Pa	Pi	Pa	Pi	Pa	Pi
1	l,4‐Benzenediol	0.453	0.009	0.467	0.067	0.309	0.032
2	Bicyclo[2.2.2]octane	0.574	0.026	0.442	0.014	0.365	0.089
3	3‐Oxo‐.beta.‐ionone	0.577	0.005	0.659	0.021	0.421	0.093
4	Heneicosane, 1‐cyclopentyl‐	0.579	0.025	0.414	0.019	0.308	0.033
5	Ethanone, 1‐(1a,2,3,5,6a,6b‐hexahydro‐3,3,6a‐trimethyloxireno[g] benzofuran‐5‐yl)‐	0.233	0.183	0.297	0.116	0.355	0.142
6	2‐Heptanone,6‐(3‐acetyl‐1‐cyclopropen‐1‐yl)‐3‐hydroxy 6‐methyl‐	0.454	0.066	0.457	0.012	0.347	0.148
7	*cis*‐9‐Hexadecenal	0.536	0.034	0.427	0.017	0.554	0.015
8	(3E)‐2‐Methyl‐4‐(1,3,3‐trimethyl‐7‐oxabicyclo[4.1.0] hept‐2‐yl)‐3‐buten‐2‐ol	0.496	0.048	0.441	0.076	0.455	0.067
9	4‐(3‐Hydroxy‐2,2,6‐trimethyl‐7‐oxa‐bicyclo[4.1.0]hept‐1‐Y1)‐but‐3‐en‐2‐one	0.445	0.071	0.279	0.088	0.455	0.067
10	4‐*tert*‐Butyl‐2‐(1‐methyl‐2‐nitro‐ethyl)‐cyclohexanone	0.08	0.026	0.289	0.096	0.478	0.05
11	5‐Caranol, (1S,3R,5S,6R)‐(‐)	0.481	0.054	0.553	0.042	0.509	0.031
12	2(4H)‐Benzofuranone, 5,6,7,7a‐tetrahydro‐6‐hydroxy‐4,4,7a‐trimethyl	0.518	0.04	0.515	0.053	0.444	0.075
13	n‐Nonadecane	0.602	0.021	0.585	0.004	0.473	0.042
14	5,7‐Octadien‐2‐one	0.658	0.014	0.383	0.028	0.425	0.055
15	4‐Hydroxy‐3,5,5‐trimthyl‐4‐(3‐oxo‐1‐butenyl)	0.4	0.093	0.305	0.156	0.312	0.173
16	3,4,5,6,7,8‐Hexahydro‐1(2H)‐naphthalenone	0.405	0.091	0.451	0.072	0.52	0.026
17	Spiro[4.5]decan‐7‐one, 1,8‐dimethyl‐8,9‐epoxy‐4‐isopropyl‐	0.152	0.1	0.317	0.146	0.48	0.049
18	Cyclohexanone, 2‐(hydroxymethyene)‐3‐methyl‐6‐(1‐methylethyl)‐	0.445	0.071	0.483	0.062	0.54	0.018
19	2‐cyclohexylethyl isobutyl ester	0.729	0.009	0.331	0.135	0.497	0.038
20	7.8‐Epoxy‐.alpha.‐ionone	0.436	0.075	0.314	0.148	0.509	0.031
21	n‐Tetradecyltrichlorosilane	0.402	0.093	0.449	0.013	0.45	0.07
22	1,2‐Benzenedicarboxylic acid, dibutyl ester	0.694	0.011	0.497	0.058	0.449	0.071
23	n‐Heneicosanea	0.602	0.021	0.585	0.004	0.565	0.012
24	(1‐Propyldecyl)cyclohexane	0.467	0.06	0.414	0.019	0.419	0.057
25	11,14,17‐Eicosatrlenoic acid methyl ester	0.887	0.004	0.804	0.006	0.56	0.013
26	3.7.11.15‐Tetramethyl‐2‐hexadecen‐1‐ol	0.828	0.005	0.458	0.07	0.3	0.182
27	8‐n‐Hexylpentadecane	0.566	0.027	0.49	0.008	0.522	0.025
28	2,4‐Dimethylicosane	0.552	0.03	0.417	0.019	0.469	0.057
29	3‐Methylhenicosane	0.676	0.013	0.44	0.014	0.493	0.04
30	1‐Octadecene	0.699	0.011	0.452	0.072	0.567	0.012
31	Dodecane. 4‐cyclohex	0.579	0.025	0.414	0.019	0.531	0.022
32	2,4‐Dimethyldocosane	0.552	0.03	0.417	0.019	0.469	0.057
33	n‐Pentacosane	0.602	0.021	0.585	0.004	0.565	0.012
34	Decane 4‐cyclohexyl‐, 4‐cyclohexyl	0.579	0.025	0.414	0.019	0.531	0.022
35	17‐Cyclohexyltritriacontane	0.467	0.06	0.414	0.019	0.539	0.019
36	EINECS 211‐124‐1	0.602	0.021	0.585	0.004	0.565	0.012
37	n‐Hexatriacontane	0.602	0.021	0.585	0.004	0.565	0.012
38	4,4,6a,6b,8a,11,11,14b‐Octamethyl 1,4,4a,5,6,6a,7,8,8a,9,10,11,12,12a,14,14b‐octadecahydro‐2H‐picen‐3‐one	0.799	0.005	0.859	0.005	0.785	0.002
39	Lup‐20(29)‐en‐3‐yl acetate	0.623	0.018	0.737	0.012	0.679	0.004
40	3,5,7‐Tris(triimethylsilox)‐2‐[3,4‐di(trimethylsiloxy)phenyl]‐4h‐1‐benzopyran‐4‐one	0.437	0.009	0.606	0.03	0.289	0.156

### 3.7. ADME/T and Drug‐Likeness Analysis

The ADME/T and drug‐likeness analysis highlighted favorable pharmacokinetic properties, including good human intestinal absorption, water solubility, and acceptable BBB permeability (Table 5). Toxicity predictions also suggested that the compounds were unlikely to induce hepatotoxicity or AMES mutagenicity, making them suitable candidates for further drug development. Lipinski′s rule of five identified 38 drug‐like compounds from 40. Given the nonpolar, lipophilic character of the GC–MS‐identified phytoconstituents, all compounds had negative log solubility values (log moles per liter) for aqueous solubility. All substances have intestinal absorption levels above 84%. Several long‐chain aliphatic hydrocarbons penetrated the BBB better. Six compounds (Compounds 5, 8, 9, 10, 17, and 20) were projected to be AMES mutagenic and deprioritized in docking analyses due to toxicity. Hepatotoxicity was anticipated for Chemicals 19 and 40: 2‐cyclohexylethyl isobutyl ester and 3,5,7‐tris(trimethylsiloxy) flavone derivative. Compounds 35 and 37 lack drug‐likeness and were excluded. Molecular docking was prioritized for the remaining compounds with good ADME/T profiles and drug‐likeness.

**Table 5:** ADME/T and drug‐likeness analysis for compounds from *Cayratia trifolia* leaf extract.

S.no.	Compound name	Absorption		Distribution		Metabolism	Excretion	Toxicity		Drug‐likeliness	Bioavailability
		Water solubility (log mol/L)	Intestinal absorption (human % absorbed)	VDss (human log L/kg)	BBB permeability (log BB)	CYP3A4 substrate	Total clearance (log mL/min/kg)	AMES toxicity	Hepatotoxicity		
1	l,4‐Benzenediol	−0.762	86.856	−0.022	−0.318	No	0.52	No	No	Yes	0.55
2	Bicyclo[2.2.2]octane	−3.906	94.121	0.631	0.83	No	0.059	No	No	Yes	0.55
3	3‐Oxo‐.beta.‐ionone	−2.964	96.923	0.108	0.153	No	0.173	No	No	Yes	0.55
4	Heneicosane, 1‐cyclopentyl‐	−7.663	88.401	0.211	1.122	Yes	1.722	No	No	Yes	0.55
5	Ethanone, 1‐(1a,2,3,5,6a,6b‐hexahydro‐3,3,6a‐trimethyloxireno[g] benzofuran‐5‐yl)‐	−2.349	98.415	0.266	0.402	No	1.019	Yes	No	Yes	0.55
6	2‐Heptanone,6‐(3‐acetyl‐1‐cyclopropen‐1‐yl)‐3‐hydroxy 6‐methyl‐	−1.774	95.802	−0.035	−0.05	No	1.368	No	No	Yes	0.55
7	*cis*‐9‐Hexadecenal	−7.144	92.839	0.458	0.809	Yes	1.883	No	No	Yes	0.55
8	(3E)‐2‐Methyl‐4‐(1,3,3‐trimethyl‐7‐oxabicyclo[4.1.0] hept‐2‐yl)‐3‐buten‐2‐ol	−3.027	93.996	0.321	0.451	No	0.974	Yes	No	Yes	0.55
9	4‐(3‐Hydroxy‐2,2,6‐trimethyl‐7‐oxa‐bicyclo[4.1.0]hept‐1‐Y1)‐but‐3‐en‐2‐one	−2.017	−2.017	−2.017	−0.013	No	1.178	Yes	No	Yes	0.55
10	4‐*tert*‐Butyl‐2‐(1‐methyl‐2‐nitro‐ethyl)‐cyclohexanone	−4.053	96.36	0.023	−0.192	No	1.301	Yes	No	Yes	0.55
11	5‐Caranol, (1S,3R,5S,6R)‐(‐)	−2.194	94.624	0.325	0.629	No	0.013	No	No	Yes	0.55
12	2(4H)‐Benzofuranone, 5,6,7,7a‐tetrahydro‐6‐hydroxy‐4,4,7a‐trimethyl	−1.641	95.691	0.086	−0.223	No	1.038	No	No	Yes	0.55
13	n‐Nonadecane	−8.565	90.015	0.642	0.996	Yes	1.96	No	No	Yes	0.55
14	5,7‐Octadien‐2‐one	−2.708	96.392	0.106	0.637	No	0.439	No	No	Yes	0.55
15	4‐Hydroxy‐3,5,5‐trimthyl‐4‐(3‐oxo‐1‐butenyl)	−1.888	96.083	−0.067	−0.043	No	0.239	No	No	Yes	0.55
16	3,4,5,6,7,8‐Hexahydro‐1(2H)‐naphthalenone	−2.498	96.461	0.319	0.31	No	1.271	No	No	Yes	0.55
17	Spiro[4.5]decan‐7‐one, 1,8‐dimethyl‐8,9‐epoxy‐4‐isopropyl‐	−3.906	96.823	0.378	0.577	Yes	0.969	Yes	No	Yes	0.55
18	Cyclohexanone, 2‐(hydroxymethyene)‐3‐methyl‐6‐(1‐methylethyl)‐	−1.232	95.472	0.029	0.373	No	0.134	No	No	Yes	0.55
19	2‐Cyclohexylethyl isobutyl ester	−4.587	95.389	0.114	0.114	Yes	1.343	No	Yes	Yes	0.55
20	7.8‐Epoxy‐.alpha.‐ionone	−2.911	97.551	0.291	0.529	No	1.147	Yes	No	Yes	0.55
21	n‐Tetradecyltrichlorosilane	−8.136	87.142	0.55	0.872	Yes	0.739	No	No	Yes	0.55
22	1,2‐Benzenedicarboxylic acid, dibutyl ester	−4.169	95.044	−0.007	−0.007	Yes	0.93	No	No	Yes	0.55
23	n‐Heneicosanea	−8.558	89.328	0.579	1.033	Yes	2.033	No	No	Yes	0.55
24	(1‐Propyldecyl)cyclohexane	−8.337	92.372	0.578	0.953	Yes	1.628	No	No	Yes	0.55
25	11,14,17‐Eicosatrlenoic acid methyl ester	−6.095	92.148	−0.616	−0.199	Yes	2.054	No	No	Yes	0.55
26	3.7.11.15‐Tetramethyl‐2‐hexadecen‐1‐ol	−7.554	90.71	0.468	0.806	Yes	1.686	No	No	Yes	0.55
27	8‐n‐Hexylpentadecane	−8.602	90.889	0.582	1.017	Yes	2.037	No	No	Yes	0.55
28	2,4‐Dimethylicosane	−8.607	90.375	0.525	1.033	Yes	1.803	No	No	Yes	0.55
29	3‐Methylhenicosane	−8.619	89.481	0.599	1.034	Yes	1.981	No	No	Yes	0.55
30	1‐Octadecene	−8.406	90.834	0.656	0.987	Yes	1.998	No	No	Yes	0.55
31	Dodecane. 4‐cyclohex	−8.258	91.426	0.591	0.591	Yes	1.595	No	No	Yes	0.55
32	2,4‐Dimethyldocosane	−8.355	89.688	0.429	1.07	Yes	1.738	No	No	Yes	0.55
33	n‐Pentacosane	−7.936	87.953	0.374	1.109	Yes	2.176	No	No	Yes	0.55
34	Decane 4‐cyclohexyl‐, 4‐cyclohexyl	−7.859	92.113	0.624	0.915	Yes	1.529	No	No	Yes	0.55
35	17‐Cyclohexyltritriacontane	−4.045	86.029	−0.485	1.331	Yes	2.004	No	No	No	0.17
36	EINECS 211‐124‐1	−7.679	87.609	0.312	1.128	Yes	2.071	No	No	Yes	0.55
37	n‐Hexatriacontane	−4.563	84.173	−0.296	1.317	Yes	2.304	No	No	No	2.17
38	4,4,6a,6b,8a,11,11,14b‐Octamethyl 1,4,4a,5,6,6a,7,8,8a,9,10,11,12,12a,14,14b‐octadecahydro‐2H‐picen‐3‐one	−6.741	96.254	0.246	0.694	Yes	−0.096	No	No	Yes	0.55
39	Lup‐20(29)‐en‐3‐yl acetate	−6.006	97.894	−0.12	0.644	Yes	0.06	No	No	Yes	0.55
40	3,5,7‐Tris(triimethylsilox)‐2‐[3,4‐di(trimethylsiloxy)phenyl]‐4h‐1‐benzopyran‐4‐one	−6.863	88.01	0.758	−1.914	Yes	−0.771	No	Yes	Yes	0.55

### 3.8. Molecular Docking Study

Molecular docking studies provided insights into the binding interactions of the Top 20 compounds with various target proteins represented in Table 6. The docking analysis revealed that the compounds exhibited strong binding affinities against human COX‐2 (PDB: 5F19) for analgesic activity, human COX‐2 (PDB: 5IKR) for anti‐inflammatory activity, and CYP2C9 (PDB: 1OG5) and Keap1 (PDB: 4L7B) for antioxidant activity. Among the top performers, lup‐20(29)‐en‐3‐yl acetate demonstrated the strongest binding across all targets, surpassing standard drugs such as diclofenac, aspirin, and ascorbic acid (AA) in several cases.

**Table 6:** Analgesic, anti‐inflammatory, and antioxidant binding affinity scores of specific compounds and standard drugs against their respective receptor.

S. no.	Compound name	Pub CID	Analgesic	Anti‐inflammatory	Antioxidant	
			5F19	5IKR	1OG5	4L7B
1	l,4‐Benzenediol	785	^∗∗^	−5.3	^∗∗^	^∗∗^
2	Bicyclo[2.2.2]octane	9235	^∗∗^	^∗∗^	−4.8	−4.4
3	3‐Oxo‐.beta.‐ionone	5363876	^∗∗^	−6.7	−6.6	−6.5
4	Heneicosane, 1‐cyclopentyl‐	23171	^∗∗^	^∗∗^	−6.5	−6
5	2‐Heptanone,6‐(3‐acetyl‐1‐cyclopropen‐1‐yl)‐3‐hydroxy 6‐methyl‐	540204	^∗∗^	−6.3	^∗∗^	^∗∗^
6	*cis*‐9‐Hexadecenal	5364643	−6.1	^∗∗^	^∗∗^	^∗∗^
7	5‐Caranol, (1S,3R,5S,6R)‐(‐)	564727	−6.2	−5.5	^∗∗^	^∗∗^
8	2(4H)‐Benzofuranone, 5,6,7,7a‐tetrahydro‐6‐hydroxy‐4,4,7a‐trimethyl	100332	^∗∗^	−5.6	^∗∗^	^∗∗^
9	5,7‐Octadien‐2‐one	129809965	^∗∗^	^∗∗^	−5.4	−5.3
10	3,4,5,6,7,8‐Hexahydro‐1(2H)‐naphthalenone	578871	−7	−6.9	^∗∗^	^∗∗^
11	Spiro[4.5]decan‐7‐one, 1,8‐dimethyl‐8,9‐epoxy‐4‐isopropyl‐	538938	−5.3	^∗∗^	^∗∗^	^∗∗^
12	Cyclohexanone, 2‐(hydroxymethyene)‐3‐methyl‐6‐(1‐methylethyl)‐	557484	−6.4	−6.5	^∗∗^	^∗∗^
13	2‐Cyclohexylethyl isobutyl ester	79253	−7.4	^∗∗^	−7.5	−7.4
14	7.8‐Epoxy‐.alpha.‐ionone	538944	−6.6	^∗∗^	^∗∗^	^∗∗^
15	1,2‐Benzenedicarboxylic acid, dibutyl ester	3026	^∗∗^	−7.1	−6.4	−6.6
16	1‐Nonadecenea	12401	^∗∗^	−6.1	−5.1	−5.1
17	n‐Heneicosanea	12403	−5.9	−6.4	−5.1	−5.3
18	11,14,17‐Eicosatrlenoic acid methyl ester	5282827	−6.2	−7	−6.2	−6.1
19	3.7.11.15‐Tetramethyl‐2‐hexadecen‐1‐ol	5366244	^∗∗^	−6.5	−6.2	−6
20	8‐n‐Hexylpentadecane	300518	−6.3	−6.8	−5.4	−5.6
21	2,4‐Dimethylicosane	558952	^∗∗^	^∗∗^	^∗∗^	^∗∗^
22	3‐Methylhenicosane	522120	−5.5	^∗∗^	−6.1	−5.4
23	1‐Octadecene	8217	−5.7	−6.1	−5.2	−5
24	Dodecane. 4‐cyclohex	15714	−6.6	^∗∗^	−6.1	−5.8
25	n‐Pentacosane	12406	−5.7	−6.6	−5.9	−5.2
26	Decane 4‐cyclohexyl‐, 4‐cyclohexyl	15713	−7	^∗∗^	−6.1	−5.4
27	17‐Cyclohexyltritriacontane	296265	−5.9	^∗∗^	^∗∗^	^∗∗^
28	EINECS 211‐124‐1	12407	−6.2	−5.9	−6.1	−5.2
29	n‐Hexatriacontane	12412	−5.8	−6.5	−6.2	−5.7
30	Lup‐20(29)‐en‐3‐yl acetate	323074	−7.8	−10.8	−10.1	−8.6
31	Diclofenac, aspirin, and ascorbic acid	3033, 2244, and 54670067	−7.4	−6.3	−5.3	−6.2

*Note:* Compounds excluded from molecular docking based on PASS prediction and ADME/T screening criteria (AMES mutagenicity, hepatotoxicity, or failure to meet drug‐likeness requirements).

∗∗ indicates the absence.

#### 3.8.1. Molecular Docking for Analgesic Activity

**Figure 4:**
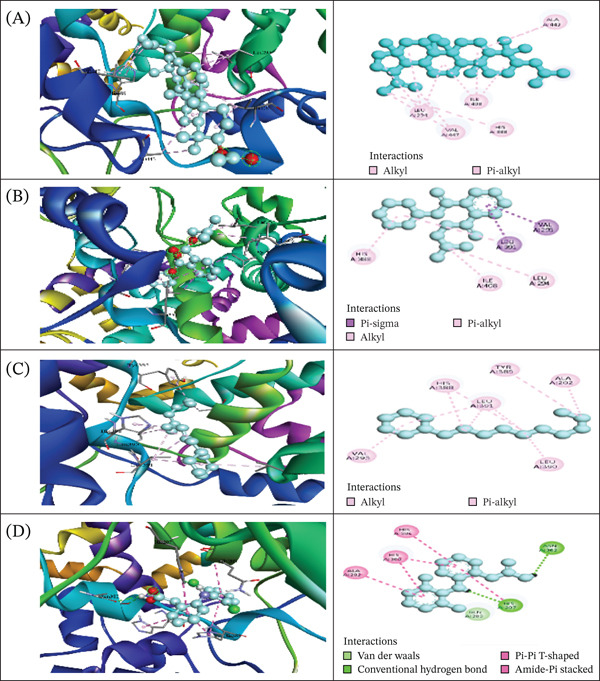
Molecular docking of chosen drugs against human COX‐2 (PDB: 5F19) to test for analgesic. (A) 3D pose and 2D interaction diagram of lup‐20(29)‐en‐3‐yl acetate. (B) 3D pose and 2D interaction diagram of 2‐cyclohexylethyl isobutyl ester. (C) 3D pose and 2D interaction diagram of 3,4,5,6,7,8‐hexahydro‐1(2H)‐naphthalenone. (D) 3D pose and 2D interaction diagram of diclofenac (standard).

The selected compounds showed analgesic potential in molecular docking against human COX‐2 (PDB: 5F19). Diclofenac, the reference drug, had a binding affinity of −7.4 kcal/mol and formed conventional hydrogen bonds with ASN A:382 and HIS A:207, pi–pi T‐shaped interactions with HIS A:386 and HIS A:388, and van der Waals contacts with ALA A:202 and GLN A:203, confirming its COX‐2 inhibitory. The phytoconstituent lup‐20(29)‐en‐3‐yl acetate has the highest binding affinity of −7.8 kcal/mol, surpassing diclofenac. This molecule paired alkyl and pi–alkyl with active site residues ALA A:443, LEU A:294, VAL A:447, HIS A:388, and ILE A:408. The presence of HIS A:388, a residue important for catalysis in the COX‐2 hydrophobic channel, suggests a favorable binding interaction, which could lead to pain relief. 2‐Cyclohexylethyl isobutyl ester had a binding affinity of −7.4 kcal/mol, equivalent to diclofenac, through pi–sigma interaction with HIS A:388 and alkyl/pi–alkyl contacts with VAL A:295, LEU A:391, LEU A:294, and ILE A:408. This suggests strong hydrophobic complementarity with the decane 4‐cyclohexyl‐bound TYR A:385, HIS A:388, ALA A:202, LEU A:391, VAL A:295, and LEU A:390 via alkyl and pi–alkyl contacts at −7.0 kcal/mol. TYR A:385 is a key residue in COX‐2′s catalytic action, supporting its analgesic potential. The findings suggest that lup‐20(29)‐en‐3‐yl acetate and 2‐cyclohexylethyl isobutyl ester exhibit the most favorable analgesic potential within the *C. trifolia* extract, demonstrating binding affinities that are either equivalent to or surpass those of diclofenac (Table 6 and Figure 4).

#### 3.8.2. Molecular Docking for Anti‐Inflammatory Activity

**Figure 5:**
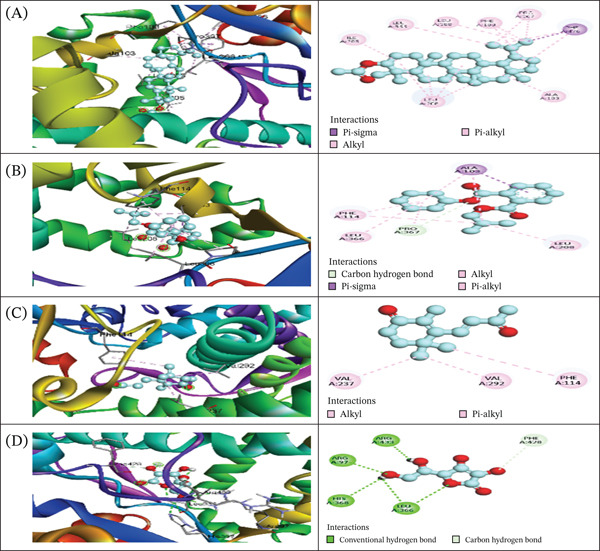
Molecular docking of chosen drugs to human COX‐2 (PDB: 5IKR) to test their ability to reduce inflammation. (A) 3D pose and 2D interaction diagram of lup‐20(29)‐en‐3‐yl acetate. (B) 3D pose and 2D interaction diagram of 1,2‐benzenedicarboxylic acid dibutyl ester. (C) 3D pose and 2D interaction diagram of 11,14,17‐eicosatrienoic acid methyl ester. (D) 3D pose and 2D interaction diagram of aspirin (standard).

The compounds under investigation exhibited anti‐inflammatory characteristics, as demonstrated by molecular docking analyses of human COX‐2 (PDB: 5IKR). Aspirin, serving as a reference, displayed a binding affinity of −6.3 kcal/mol. Notably, lup‐20(29)‐en‐3‐yl acetate, one of the phytoconstituents, displayed a significantly greater binding affinity of −10.8 kcal/mol, thereby exceeding the affinity observed for aspirin. This particular molecule established significant alkyl and pi–alkyl interactions with LEU A:93, TYR A:115, VAL A:89, VAL A:116, VAL A:349, LEU A:531, and LEU A:352, alongside hydrogen bonds with SER A:530 and TYR A:385, which are two of the most crucial catalytic residues within the COX‐2 active site. The interference of SER A:530 and TYR A:385 with the COX‐2 catalytic pathway strongly supports its anti‐inflammatory potential. Furthermore, mild steric strain, a characteristic often observed in large natural product scaffolds that does not impede biological activity, may lead to bump interactions with TYR A:355, VAL A:116, ARG A:120, and ALA A:527 (Table 6 and Figure 5).

#### 3.8.3. Molecular Docking for Antioxidant Activity

**Figure 6:**
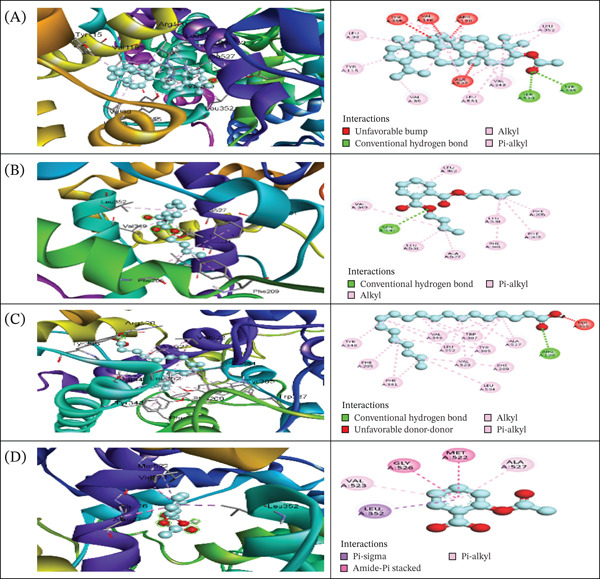
Molecular docking of chosen compounds against CYP2C9 (PDB: 1OG5) to see if they have antioxidant properties. (A) 3D pose and 2D interaction diagram of lup‐20(29)‐en‐3‐yl acetate. (B) 3D pose and 2D interaction diagram of 2‐cyclohexylethyl isobutyl ester. (C) 3D pose and 2D interaction diagram of 1,2‐benzenedicarboxylic acid dibutyl ester. (D) 3D pose and 2D interaction diagram of ascorbic acid (standard).

**Figure 7:**
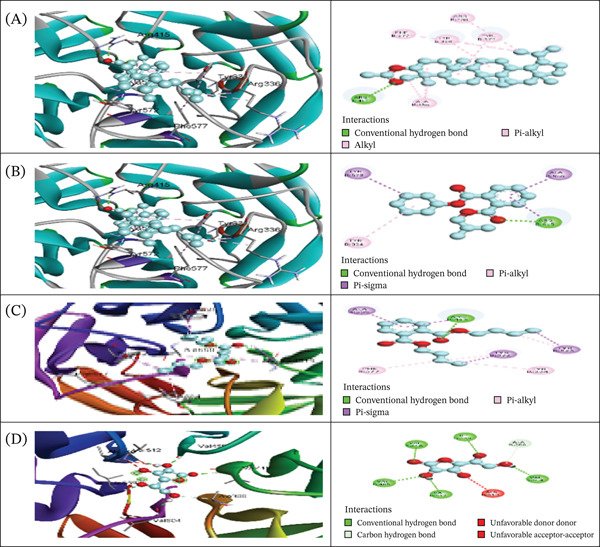
Molecular docking of chosen compounds against Keap1 (PDB: 4L7B) to assess antioxidant activity. (A) 3D pose and 2D interaction diagram of lup‐20(29)‐en‐3‐yl acetate. (B) 3D pose and 2D interaction diagram of 2‐cyclohexylethyl isobutyl ester. (C) 3D pose and 2D interaction diagram of 1,2‐benzenedicarboxylic acid dibutyl ester. (D) 3D pose and 2D interaction diagram of ascorbic acid (standard).

We employed molecular docking to investigate the antioxidant capacity of the screened compounds against CYP2C9 (PDB: 1OG5) and Keap1 (PDB: 4L7B) using AA as the reference compound (−5.3 and −6.2 kcal/mol, respectively). Lup‐20(29)‐en‐3‐yl acetate exhibited superior binding to CYP2C9, as indicated by a binding energy of −10.1 kcal/mol, when compared to AA. This compound engaged in a pi–sigma interaction with the active site residue PHE A:476 of CYP2C9. Furthermore, it formed pi–alkyl interactions with LEU A:388, PHE A:100, PRO A:367, and LEU A:366, alongside alkyl contacts with ILE A:205, LEU A:208, and ALA A:103. The significance of PHE A:476 is underscored by its critical role in substrate recognition and binding within the CYP2C9 hydrophobic pocket. Moreover, lup‐20(29)‐en‐3‐yl acetate showed a binding affinity of −8.6 kcal/mol for Keap1 (PDB: 4L7B), which was better than AA. This compound established a consistent hydrogen bond with ARG B:415, a critical element of the Keap1 Kelch domain that regulates Nrf2 protein binding. Furthermore, pi–alkyl interactions with TYR B:334, B:572, and ARG B:336 were enhanced, alongside alkyl interactions with PHE B:577 and ALA B:556. The direct interaction between ARG B:415 and Keap1 could potentially disrupt the Nrf2–Keap1 interaction. This would then allow Nrf2 to move into the nucleus, which would increase the production of natural antioxidant enzymes like HO‐1 and NQO1. Therefore, lup‐20(29)‐en‐3‐yl acetate shows a strong attraction to both antioxidant target proteins and important amino acids, making it the most suitable candidate for antioxidant properties (Table 6 and Figures 6 and 7).

## 4. Discussion

Distinct pharmacological effects are caused by distinct chemical groupings. The potential pharmacological action of the crude extract is shown by the identification of various chemical groups [30]. The crude methanol extract of *C. trifolia* leaves contained triterpenoids and phytosterols, which may have biological properties like antiseptic, anthelmintic, and antibacterial agents. The MECT also contained flavonoids, which have been shown to have biological properties like antiviral, antioxidative, antiulcer, and anti‐inflammatory responses.

The leaves contained a small amount of saponins. In some animal species, dietary saponins decreased plasma cholesterol levels, inflammation, chest discomfort, stenocardia, and cardiac asthma [31]. It may possess cytotoxic, hemolytic, antitumor, antibacterial, and antiparasitic qualities [32]. According to their biological activity, the tannins in the crude methanol extract were tested as antitussive, astringent, anticancer, and antioxidant agents. Additionally, it included alkaloids, which have been utilized as analgesics, antispasmodics, anticancer, antimalaria, and antitussive activity [33]. MECT also tested positive for carbohydrates, fats, and fixed oils. Although these constituents are not usually considered primary bioactive markers, they may contribute to the plant′s physiological functions and may also reflect its supplementary nutritional value.

In general, the methanolic extract, being the parent extract, is expected to retain a wider range of phytochemicals with diverse polarity, whereas the n‐hexane fraction is likely to be enriched predominantly with nonpolar or moderately nonpolar constituents. Therefore, differences in the biological activities of MECT and NHCT may partly be attributed to differences in their phytochemical composition. However, this interpretation should be made cautiously, as the present study relied on previously published GC–MS data in which only the methanolic extract was characterized, while corresponding GC–MS data for the n‐hexane fraction were unavailable.

One recognized method for evaluating plant extracts′ antioxidant capacity is DPPH free radical scavenging. The benefit of this approach is that it gives DPPH enough time to react with even weak antioxidants and permits DPPH to react with the entire sample. The higher the percentage of scavenging action, the stronger the antioxidant capacity of the extracts in the current study, which uses the DPPH method to measure the antioxidant properties of the examined extracts. Conversely, the extracts′ antioxidant activity increases with a lower IC_50_ value. At the maximum concentration in this investigation, the scavenging effect of the reference standard AA was 92.44%. AA had a substantially lower IC_50_ value (1.89 *μ*g/mL). This extract contains compounds that are essential in minimizing oxidation and supporting overall health. Findings indicated that the crude MECT leaves are a highly effective free radical scavenger, surpassing AA at the highest concentration, though it did not exceed AA′s scavenging percentage and exhibited a concentration‐dependent effect. Various flavonoid constituents within the extract may contribute to its antioxidant and free radical scavenging activities through mechanisms such as blocking, interfering with, or inhibiting enzymes responsible for reactive oxygen species production; neutralizing free radicals; and chelating transition metals to deactivate harm [34]. The antioxidant properties of the extract are likely due to the combined effects of these compounds. More investigation is essential for gaining a more comprehensive insight into the mechanisms involved with the DPPH radical and potential antioxidants.

The in vitro anti‐inflammatory activity has been measured by the stability of the HRBC membrane, or the inhibition of HRBC membrane rupture caused by hypotonic conditions. MECT and diclofenac sodium were tested at different concentrations to determine the percentage of membrane stabilization. At varying doses, MECT was successful in preventing hypnocity‐induced hemolysis of HRBC. This anti‐inflammatory action was due to the occurrence of different phytochemicals, namely, flavonoids, triterpenoids, saponins, and alkaloids. At the highest dose, the highest inhibition was 87.46%. Membrane stabilization/protection increases, and membrane hemolysis decreases as concentration rises. As a result, the extracts′ anti‐inflammatory properties varied with concentration. The absorbance of hemoglobin was measured in the current study using the HRBC membrane stabilization method. The breakdown of the erythrocyte membrane resulted in the release of hemoglobin. Less absorbance was observed in the spectrometer data as a result of membrane stabilization. By preventing lysis of erythrocyte membrane caused by hypotonicity, the methanolic leaf extract of *C. trifolia* demonstrated a membrane‐stabilizing effect. Since the lysosomal membrane and the erythrocyte membrane are similar, the extracts may also stabilize the lysosomal membrane. By stopping the discharge of lysosomal substances from activated neutrophils, including bacterial enzymes and proteases, that enhance inflammation and injury, stabilization of lysosomal membranes plays a vital part in alleviating inflammation [35]. Significant membrane‐stabilizing properties demonstrated by MECT raised the possibility that the anti‐inflammatory action seen in this investigation was caused by the extract′s ability to prevent the release of phospholipases, causing the release of inflammatory mediators.

Data derived from the carrageenan‐induced paw inflammation model, considered a reliable method to evaluate short‐term anti‐inflammatory action, showed that the control group showed no reduction in paw edema. However, the reference standard, diclofenac sodium, significantly reduced the edema starting from the second hour. When compared to the control, the MECT, along with the NHCT, demonstrated a dose‐dependent reduction in edema. These findings lead to the conclusion that the extracts have a potent anti‐inflammatory effect, in comparison with standard diclofenac. The occurrence of flavonoids, glycosides, and tannins in the extract has been associated with anti‐inflammatory activities [36]. Therefore, these compounds can be accountable to the observed anti‐inflammatory effects of the tested extracts. However, further investigation is required to explore the fundamental mechanism of the extracts and to detect and isolate the active constituents that may contribute to their potential effects.

The abdominal writhing assay triggered by acetic acid serves as a screening method for assessing the pain‐relief substances. In the current study, writhing caused by acetic acid has been utilized to screen the peripheral antinociceptive effect [37], which uses a diluted form of acetic acid to induce a writhing response in laboratory animals [38]. In this experiment, a lower number of writhes in mice and a higher level of inhibition of writhing indicated a stronger pain‐relieving effect at the peripheral level. The control group exhibited an increased number of writhings, while the reference standard showed the fewest writhing episodes and a significant 60.19% inhibition. MECT produced significant peripheral analgesic activity (36.89%), but its effect remained lower than that of diclofenac sodium, while NHCT also showed a significant inhibitory effect (21.84%). All the extracts examined in this study demonstrated a significant, dose‐dependent effect as antinociceptive agents. The pain‐relieving effects of this plant are attributed to its content of alkaloids, flavonoids, tannins, and terpenoids. Additional research is needed to assess the antinociceptive effects using other methods and to explore the underlying mechanisms and pathways responsible for these effects. During the tail immersion test, the extract enhanced resistance to stress, suggesting the potential association with higher centers. The nociception‐inhibiting effect of the methanolic leaf extract of *C. trifolia* at a concentration of 400 mg/kg was significant, with reaction time extensions in the tail immersion model. Both MECT and NHCT produced significant analgesic effects in peripheral and central models; however, MECT showed stronger peripheral activity in the writhing test, whereas NHCT exhibited a relatively stronger central analgesic response in the tail immersion test. Analysis indicates that the extracts of *C. trifolia* possess analgesic properties through both central and peripheral pathways, as evidenced by significant increases in reaction time to thermal stimuli and inhibition of writhing induced by acetic acid.

An initial assessment of the compounds screened from *C. trifolia* leaf extract for pharmacological potential was conducted using PASS prediction, which highlighted the Top 20 candidates exhibiting high probability values (Pa) for analgesic, anti‐inflammatory, and antioxidant activities. ADME/T profiling assessed their drug‐likeness and safety, indicating favorable oral bioavailability, low predicted toxicity, and acceptable pharmacokinetic properties. Compounds with high projected activity and interesting pharmacological profiles were efficiently prioritized using the combination of PASS and ADME/T analysis, which streamlined the selection process for molecular docking investigations. All together, these results show the successful discovery of bioactive phytochemicals with promising pharmacological potential and tolerable safety profiles, bolstering their selection for in silico PASS and ADME/T screening [39].

Direct GC–MS profiling of the prepared extracts was not performed because this study was conducted within the limited time and funding scope of a thesis project, and the available extract was primarily used for the biological assays. Therefore, previously published GC–MS data were used as a literature‐based reference for in silico compound selection. However, phytochemical composition may vary with geography, season, extraction conditions, and instrumental settings; thus, the reported compounds should be regarded as literature‐guided candidates rather than the definitive composition of the studied extract batch.

The therapeutic effects of *C. trifolia* compounds, as elucidated through molecular docking studies, are mediated by their interactions with key proteins involved in inflammation, oxidative stress, and pain signaling, providing insight into their potential cellular mechanisms. The compounds, namely, lup‐20(29)‐en‐3‐yl acetate and 2‐cyclohexylethyl isobutyl ester, demonstrated substantial binding affinities for essential therapeutic targets. Lup‐20(29)‐en‐3‐yl acetate showed the highest binding affinity for human COX‐2 (PDB: 5F19; −7.8 kcal/mol) in terms of pain relief, more than diclofenac (−7.4 kcal/mol), through interactions with HIS A:388, ALA A:443, LEU A:294, VAL A:447, and ILE A:408. The compound had a very strong binding affinity for human COX‐2 (PDB: 5IKR; −10.8 kcal/mol), forming important hydrogen bonds with SER A:530 and TYR A:385. This stopped the production of prostaglandins and reduced the inflammatory response [40]. This compound may reduce oxidative stress by activating PHE A:476 and blocking Keap1 at ARG B:415. This would increase the Nrf2‐mediated production of the antioxidant enzymes HO‐1 and NQO1, which work against CYP2C9 (PDB: 1OG5; −10.1 kcal/mol) and Keap1 (PDB: 4L7B; −8.6 kcal/mol). 2‐Cyclohexylethyl isobutyl ester worked very well as a pain reliever, just like diclofenac (−7.4 kcal/mol) against COX‐2 (PDB: 5F19). The results suggest that the phytoconstituents of *C. trifolia* confer therapeutic advantages by modulating essential pathways linked to inflammation, oxidative stress, and pain. Further experimental validation is necessary to corroborate these findings [41]. The analgesic properties are attributed to their ability to inhibit COX‐2, reducing the production of pain‐inducing prostaglandins. Additionally, these compounds may modulate nociceptive pathways by influencing the release of pain‐associated molecules like bradykinin and substance P. The docking results suggest that these compounds may alleviate pain both peripherally and centrally, making them effective for treating inflammatory and chronic pain.

Overall, the molecular docking study indicates that *C. trifolia* compounds exert their therapeutic effects by modulating key cellular pathways involved in inflammation, oxidative stress, and pain, providing a basis for future therapeutic development. Further experimental validation is needed to confirm these findings.

## 5. Conclusion

The present study demonstrates that the MECT and NHCT leaves possess notable antioxidant, anti‐inflammatory, and analgesic activities, supported by phytochemical screening, in vitro and in vivo assays, and complementary in silico analyses. Among the investigated compounds, lup‐20(29)‐en‐3‐yl acetate showed the strongest docking performance against human COX‐2 (PDB: 5F19 and 5IKR), CYP2C9 (PDB: 1OG5), and Keap1 (PDB: 4L7B), while 2‐cyclohexylethyl isobutyl ester also demonstrated promising analgesic‐related binding affinity. Nevertheless, the findings should be interpreted with caution because the study did not include direct GC–MS profiling of the investigated extract batch, phytochemical screening of NHCT, or isolation and quantification of the active constituents, and the docking analysis provides preliminary supportive evidence rather than definitive mechanistic confirmation. Therefore, future studies should focus on direct chemical profiling, isolation of bioactive compounds, validation through cellular and molecular assays, and further safety assessment. Overall, *C. trifolia* may be considered a promising natural source of bioactive molecules for future antioxidant, anti‐inflammatory, and analgesic drug development.

## Author Contributions

Nasrin Jubayda, Md. Tanveer Ahsan, and Mohammad Arman were responsible for the conceptualization of the study. Data curation was carried out by Nasrin Jubayda and Md. Tanveer Ahsan. Formal analysis was performed by Nasrin Jubayda, Mohammad Arman, and Md. Kaisar Alam. Funding acquisition was managed by Nasrin Jubayda and Md. Tanveer Ahsan. The investigation was conducted by Md. Tanveer Ahsan, Mohammad Arman, and Md. Kaisar Alam. Methodology development was undertaken by Nasrin Jubayda and Mohammad Arman, while project administration was handled by Md. Tanveer Ahsan. Resources were provided by Mohammad Arman, and supervision was performed by Md. Tanveer Ahsan. Validation was completed by Md. Tanveer Ahsan, Mohammad Arman, and Md. Kaisar Alam, with visualization contributed by Md. Tanveer Ahsan, Mohammad Arman, and Md. Kaisar Alam. The in silico analysis was performed by Tanbirul Azim Maharaj and T. S. Farah Mousumi. The original draft was written by Nasrin Jubayda, Md. Tanveer Ahsan, and Md. Kaisar Alam, and the review and editing were completed by Mohammad Arman.

## Funding

This study was funded by the Ministry of Science and Technology, Government of the People′s Republic of Bangladesh (10.13039/501100008804) and the National Science and Technology Fellowship 2022–2023.

## Ethics Statement

The study was reviewed and was authorized by the Pharmacy Department′s P&D Committee (Pharm‐P&D‐61/08‐16–122) at the International Islamic University Chittagong, Bangladesh.

## Conflicts of Interest

The authors declare no conflicts of interest.

## Supporting information


**Supporting Information** Additional supporting information can be found online in the Supporting Information section. File S1: Chemical structures of the Top 3 docked compounds with their binding scores for analgesic activity against COX‐2 (PDB: 5F19), anti‐inflammatory activity against COX‐2 (PDB: 5IKR), and antioxidant activity against CYP2C9 (PDB: 1OG5) and Keap1 (PDB: 4L7B).

## Data Availability

All the data related to the manuscript have been mentioned.
