# Supplementary material for: Integrated Phytochemical, Pharmacological, and In Silico Evaluation of the Methanolic Extract of Cayratia trifolia Leaves: Antioxidant, Anti‐Inflammatory, and Analgesic Activities
Source: ScientificWorldJournal. 2026 Jun 18;2026:8390762. doi: 10.1155/tswj/8390762 (PMC13277625; doi:10.1155/tswj/8390762)
Supplement: Supplementary file 1 — Supporting Information Additional supporting information can be found online in the Supporting Information section. File S1: Chemical structures of the Top 3 docked compounds with their binding scores for analgesic activity against COX‐2 (PDB: 5F19), anti‐inflammatory activity against COX‐2 (PDB: 5IKR), and antioxidant activity against CYP2C9 (PDB: 1OG5) and Keap1 (PDB: 4L7B). [file TSWJ-2026-8390762-s001.docx]

1. Analgesic activity

| Compound Name | Structure | Binding Score |
| --- | --- | --- |
| LUP-20(29)-EN-3-YL ACETATE |  | -7.8 |
| 2-cyclohexylethyl isobutyl ester |  | -7.4 |
| 3,4,5,6,7,8-HEXAHYDRO-1(2H)-NAPHTHALENONE |  | -7.0 |
| Chemical structures of the top three compounds for analgesic activity (PDB: 5F19) | | |

1. Anti-inflammatory activity

| Compound Name | Structure | Binding Score |
| --- | --- | --- |
| LUP-20(29)-EN-3-YL ACETATE |  | -10.8 |
| 1,2-Benzenedicarboxylic acid, dibutyl ester |  | -7.1 |
| 11,14,17-EICOSATRIENOIC ACID. METHYL ESTER |  | -7.0 |
| Chemical structures of the top three compounds for anti-inflammatory activity (PDB: 5IKR) | | |

1. Antioxidant activity

| Compound Name | Structure | Binding Score |
| --- | --- | --- |
| LUP-20(29)-EN-3-YL ACETATE |  | -10.1 |
| 2-cyclohexylethyl isobutyl ester |  | -7.5 |
| 1,2-Benzenedicarboxylic acid, dibutyl ester |  | -6.6 |
| Chemical structures of the top three compounds for antioxidant activity (PDB: 1OG5) | | |

| Compound Name | Structure | Binding Score |
| --- | --- | --- |
| LUP-20(29)-EN-3-YL ACETATE |  | -8.6 |
| 2-cyclohexylethyl isobutyl ester |  | -7.4 |
| 1,2-Benzenedicarboxylic acid, dibutyl ester |  | -6.6 |
| Chemical structures of the top three compounds for antioxidant activity (PDB: 4L7B) | | |
